# Quality and Health Risk Assessment of Groundwaters in the Protected Area of Tisa River Basin

**DOI:** 10.3390/ijerph192214898

**Published:** 2022-11-12

**Authors:** Thomas Dippong, Maria-Alexandra Resz

**Affiliations:** 1 Faculty of Science, Technical University of Cluj-Napoca, 76 Victoriei Street, 430122 Baia Mare, Romania; 2 INCDO-INOE 2000, Research Institute for Analytical Instrumentation, 67 Donath Street, 400293 Cluj-Napoca, Romania

**Keywords:** non-carcinogenic risk, heavy metal pollution indices, drinking water sources, water typology

## Abstract

This study was conducted in order to assess the chemistry (41 metalloids and heavy metals and 16 physico-chemical indicators) of groundwater sampled from the protected area of the Tisa River Basin during the months of 2021. Pollution indices were used in order to determine the potential metal pollution level. Consequently, a non-carcinogenic risk assessment of metal through the ingestion of water was done. The results indicated general contamination with ammonium, chloride, iron, and manganese. The samples were rich in Cu, Mg, and Pb, but lower than the maximum limits. Significant correlations were noticed between Al-Fe, Mn-Fe, Mn-Ni, and Cr-Zn, as well as the metal content and pollution index scores. The metal pollution indices indicated three pollution levels (low, medium, and high) based on the metal content and standards regarding the water quality used for drinking purposes. The pollution indices scores ranged from 1.52–41.2. A human health risk assessment indicated no potential non-carcinogenic risk for the studied metals through the consumption of groundwater. The results of three different tools (chronic daily intake, hazard quotient, and hazard index) were below the critical value, except for the aluminium in two samples. This study is one of the first attempts to evaluate the quality of groundwater sources associated with the human health risks of the studied metals from the Tisa River Basin protected area. Based on this research, strategies for managing and controlling the risks can be developed.

## 1. Introduction

One of the most significant and valuable natural resources on Earth is represented by water, especially water used as drinking water source [1]. It is estimated that 1.8 billion people (28% of the world population) use untreated water, while 1.2 million (18% of the world population) use water sources with high sanitary risks [2]. The terrestrial ecosystems depend on groundwater in different ways, including seasonally or continually [3]. Groundwater, particularly alluvial aquifers, is a significant source of drinking water and minerals, especially in developing countries with rural and semi-urban populations; therefore, quality assessment and continued and extensive monitoring are of serious concern. Water contains many minerals, nutrients, and dissolved substances. Unfortunately, all water sources, including groundwater, are contaminated and altered due to the presence of toxic contaminants and elements entering the water systems through the hydrological cycle, which also implies continuous degradation [1].

The quality of groundwater depends on the geological structure of the area. The natural pollution (including biological processes, such as weathering, precipitation, ion exchange, and dissolution) and anthropogenic pollution (including industrial and agricultural activities) influence and alter the chemical composition [4,5]. The balance and functionality of groundwater sources depend on the physico-chemical and microbiological activities of the water system. Climatic factors (such as air humidity, precipitation, and temperature) are responsible for the groundwater supply [6]. However, the action of factors is influenced by soil, vegetation, the hydro-physico characteristics of geological formations, and surface leaks [7]. Hydrologic factors (including surface stagnant waters, shallow runoffs on slopes, and total leakage from the hydrographic network) influence the supply and groundwater regime. This influence is associated with the interactions between the balance elements of the drainage basin and groundwater. Hydrogeochemical processes (such as cation exchange, mineral dissolution, groundwater mixing, transpiration, and evaporation) influence and control the characteristics of groundwater sources [8].

Heavy metals are considered significant pollutants due to their high bioaccumulation (in tissues), biomagnification (through the food chain), toxicity, and persistency if they exceed the maximum allowable concentrations (MACs), which causes diverse diseases, such as liver crisis, skin irritation, and kidney and cardiovascular affections [9]. Epidermal absorption, inhalation, and food and water ingestion are the main sources of heavy metal accumulation in human and animal bodies [10]. Sources of heavy metals in the water systems are represented by natural processes, such as the weathering of rocks (also known as the geographic heterogeneity) rich in minerals and anthropogenic factors, such as industrial wastes, sewage leachates, municipal waste disposal, and the inappropriate use of pesticides and fertilizers used in agriculture [1,11,12].

Lately, the quality of groundwater, pollution sources, and health risks are being investigated worldwide. For example, in Nigeria, China, Romania, and India, the results indicated insecure drinking water sources led to health issues, especially in infants, particularly due to anthropogenic actions [13,14,15]. The quality of the groundwater and its risks can also be evaluated using diverse mathematical instruments; for example, pollution indices, quality index, or health risk indices.

In the present study, a series of chemical parameters were determined and assessed from groundwater samples collected from the protected site of the Tisa River Basin, such as the pH, the electrical conductivity, the oxidation-reduction potential, the temperature, the saturation level of oxygen, the turbidity, the total hardness, the content of dissolve oxygen, the total dissolved solids, the content of nutrients, and the presence of heavy metals. All chemical indicators are significant in the global assessment of water quality. Water has a crucial role in maintaining the balance of aquatic and terrestrial ecosystems in the Tisa River protected areas and generally in maintaining the functions of ecosystems as support for biodiversity. The anthropogenic pressures on the available natural resources need to be diminished or even stopped in order to assure balance between the conservation of the biodiversity in both protected Tisa River sites and the needs of inhabitants. This balance has to relate to the principles of sustainable development principles to meet the long-term needs of current generations without compromising those of future generations. Accordingly, comprehensive pollution indices were applied for the first time and analyzed in order to determine the potential pollution level of the waters and the human health risk indices to then evaluate the non-carcinogenic risks associated with the studied contaminants. The water typology of samples was also analyzed by using four different diagrams (Piper, Gibbs, Stiff, and Schoeller). The obtained results produce significant data and evidence regarding the groundwater from protected sites in the Tisa River Basin (which is also a source of drinking water) and better risk management and pollution prevention.

## 2. Materials and Methods

### 2.1. Study Area Location

**Figure 1:**
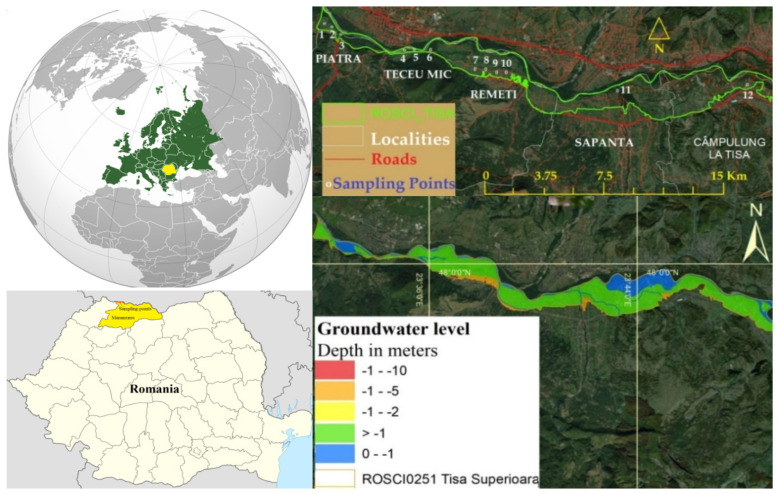
Sampling points and the aquifer levels from Tisa Superioara.

Natura 2000 ROSCI0251 the Tisa Superioara site contains the alluvial plain and terraces from the left shore of the Tisa River (Figure 1), which is part of the upper course or the river and represents the border between Romania and Ukraine (cutting the Maramures Depression from east to west). In the Tisa meadow, numerous habitats have formed, including Piatra, Teceu Mic, Remeti, Sapanta, and Campulung de Tisa. The anthropization degree is high, while the anthropogenic pressure is moderate to critical, and it manifests under a variety of forms: localities, agricultural activities, animal husbandry, traffic, abandoned household and construction wastes, fire vegetation, clearance, and sand and gravely recovery. The groundwater sources from the study area are at low and deep depths. The groundwater bodies are made of gravel and boulders situated in plain areas and in the alluvial plains of the rivers.

### 2.2. Sampling and Preservation

During each month in 2021, a sample was taken from 12 dug wells located on properties in five localities situated in a protected area (Figure 1). The dug wells were open on the property of the inhabitants and carved at a depth of 4–6 m with an 80 cm diameter. They are made of cement-asbestos or rock tubes and fitted with a pulley system for water use.

The sampling was performed according to standard procedures (SR ISO 5667–23:2011; SR ISO 5667–3:2013). Clean high-density polyethylene bottles, rinsed with the water sample, were directly inserted at 10 cm depth into the groundwater, allowing them to fill without air. The physico-chemical parameters were determined in situ using portable equipment. For the trace metals content analysis, samples were acidified with 65% nitric acid until pH 1–2 to prevent precipitation and retention of metals on the walls of the sampling bottles. All samples were preserved by refrigeration in thermal boxes protected from the sunlight and transported to the laboratory for analysis within 24 h. Three water samples were taken at each sampling station.

### 2.3. Experimental Methods

Groundwater samples were studied in order to evaluate the chemical components and therefore their quality. A number of 32 heavy metals (Ag, As, Au, Bi, Cd, Co, Cr, Cs, Cu, Fe, Ga, Ge, Hf, In, Ir, Mn, Mo, Nb, Ni, Pb, Pd, Pt, Rb, Rh, Sn, Sr, Te, Ti, Tl, V, Zn, Zr), nutrients (NH_4_^+^, NO_2_^−^, NO_3_^−^, Cl^−^, PO_4_^3−^, SO_4_^2−^, CO_3_^2−^, HCO_3_^−^, Al, Ba, Be, Ca, K, Li, Mg, Na, and Sr) and physico-chemical indicators (pH, electrical conductivity- EC, oxidation-reduction potential- ORP, total hardness- H_t_, the content of dissolve oxygen- DO, turbidity- T, and total dissolved solids- TDS) were analyzed using the 12 samples collected during every month of 2021. The oxidative-reduction potential, dissolved oxygen, oxygen saturation, and the pH were analyzed according to SR ISO 10523/2012, SR EN ISO 5814:2013, and SR ISO 10523/2012. Additionally, the nitrogen compounds were analyzed according to SR ISO 6777/2002 for NO_2_, SR ISO 7150-1/2001 for NH_4_^+^, and SR ISO 7890-3/2000 for NO_2_ by using a portable equipment Hach Lange HQ40d. The CO_3_^2−^, HCO_3_^−^, and total harness were determined according to the American Public Health Association APHA (1999) and SR ISO 6059-2008. The anion content was determined according to ISO 9297-2001 and STAS 3265-86 using a Hach Lange SL1000 portable equipment and a Perkin Elmer Lambda 25 spectrophotometer. The metal content was analyzed using mass spectrometry with the help of a Perkin Elmer NexlON 300S inductively coupled plasma mass spectrometer, according to SR EN ISO 15586-2004. The samples were prepared via acidification with 65% HNO_3_ (Merck), followed by heating at a controlled temperature, pressure, and filtering with 0.45 µm acetate cellulose filters. The methods were verified by analyzing internal standards, blanks, and triplicates, with a recovery ranging from 89% to 105%. The equipment was calibrated with standard solutions traceable to SRM from NIST Certipur.

### 2.4. Statistics and Water Typology

The results are represented as the mean value in 2021 with the standard deviation calculated based on the values obtained in the 12 months of 2021.

The water typology was determined by using different plots (Piper, Gibbs, Stiff, and Schoeller). Piper was based on the amounts of the major cations (Ca^2+^, Mg^2+^, Na^+^, and K^+^) and anions (Cl^−^, CO_3_^2−^, HCO_3_^−^, and SO_4_^2−^), indicating the various types of water [16]. Gibbs was plotted based on the total dissolved solids content and the ratios of Na^+^/(Na^+^ + Ca^2+^) and Cl^−^/(Cl^−^ + HCO_3_^−^) [17]. With the help of the Gibbs plot, the main chemical processes in the groundwater resources were assessed, such as the interaction between the water and rocks, the evaporation-crystallization process, and the atmospheric precipitation [18,19,20]. The Gibbs plot is based on the ratio of two main ions—Cl^−^/(Cl^−^ + HCO_3_^−^), Na^+^/(Na^+^ + Ca^2+^) —related to the total dissolved content [18,19,20]. The Stiff and Schoeller plots are the graphical representations of the major cations and the anion content in the water samples [21].

For the current study, the free versions of XLStat (Addinsoft, New York, DC, USA), Microsoft Excel (version 2210, Microsoft Corporation, Washington, VA, USA), and AqQa, GW_Chart version 1.29 software (US Geological Survey, Reston, VA, USA) were used for statistical calculations and obtaining the diagrams of interest. For the calculations, the mean value of results obtained in all studied months in 2021 was used.

### 2.5. Pollution Indices

The pollution status of heavy metals can be assessed by applying pollution indices. Two of the most commonly used heavy metal pollution indices are PI (Pollution Index) and HEI (Heavy metal Evaluation Index).

#### 2.5.1. Pollution Index (PI)

The suitability of water for human consumption and the overall quality of water were evaluated using the PI [22,23]. PI was calculated based on several chemical parameters (heavy metals), guideline values, and specific subindices. The applied guideline values followed the regulations established by the World Health Organization for the quality of water. PI was calculated with the help of the following equation (Equation (1)):

(1) $$PI=\frac{\sum_{i=1}^{i=n}\left(Q_{i}\times W_{i}\right)}{\sum_{i=1}^{i=n}W_{i}}$$

where *Q_i_* is the subindex of the *i*th chemical indicator or the ration between the monitored value of the heavy metal and the guideline value (*Q_i_* = (*M_v/_G_v_*) × 100); *W_i_* represents the unit weight of the *i*th chemical parameter (*W_i_* = 1/*G_v_* for each heavy metal); *n* is the total number of the considered heavy metals; and *M_v_* and *G_v_* are the monitored and guideline values of the chemical parameters [23]. The *PI* scores classify the waters into one of the three pollution level categories. *PI* < 15 indicates a low pollution level, while 15 < *PI* < 30 indicate a medium pollution level, and *PI* > 30 indicates a high pollution level [22].

#### 2.5.2. Heavy Metal Evaluation Index

*HEI* is an elementary method reported strictly at the guideline values based on the heavy metal content. This method was applied by using the following equation (Equation (2)), according to Edet and Offiong [22]:

(2) $$HEI=\sum_{i=1}^{n}\frac{M_{v}}{G_{v}}$$

where *M_v_* is the monitored value of the studied heavy metal and *G_v_* is the applied guideline value for the heavy metal [24]. The used guideline values were generally those established by national and international legislations [25,26] According to Gharderpoori [24], there are three classes of pollution: low (*HEI* < 10), medium (10 < *HEI < 20*), and high pollution (*HEI* > 20).

#### 2.5.3. Health Risk Assessment

In order to assess the health risk (non-carcinogenic) based on the oral intake of water contaminated with metals, different tools can be used, such as the chronic daily intake (*CDI*), the hazard quotient (*HQ*), or the hazard index (*HI*) [27,28,29]. The indices were calculated by using the following equations (Equations (3)–(5)):

(3) $$CDI=\frac{C\times IR\times EF\times ED}{BW\times AT}$$

(4) $$HQ=\frac{CDI}{RfD}$$

(5) HI=∑HQ

where *C* represents the metal concentration (mg/L) and *IR*, *ED*, and *EF* are the ingestion rate (2 L/day), exposure duration (30 years), and frequency (365 days/year). *BW* and *AT* are the body weight (70 kg) and the average exposure time (365 × ED). *RfD* represents the reference dose for each contaminant according to the Integrated Risk Information Systems [30]. The reference doses for each chemical are 0.004 mg/kg As, 1.5 mg/kg Cr, 0.0005 mg/kg Cu, 0.14 mg/kg Mn, 0.02 mg/kg Ni, 0.004 mg/kg Pb, 0.3 mg/kg Zn and 0.00143 mg/kg Al [30]. The *HQ* and *HI* scores indicate whether the studied water presents non-carcinogenic risks to contaminants if it is used for drinking purposes. In this case, *HQ* > 1.0 and *HI* > 1.0 indicate waters that pose health risks due to the analyzed metals, while *HQ* < 1.0 and *HI* < 1.0 indicate no risks.

## 3. Results and Discussion

### 3.1. Water Quality Characterization and Effect on Human Health

The physico-chemical characteristics of the studied groundwater samples are presented in Table 1. According to the pH values, the waters are weak, basic, and neutral, indicating the presence of weak base salts in the soil near the water system [31]. Also, the decrease of the pH is related to the increase of CO_2_, while the increase of the pH is associated with the increase of the alkalinity and HCO_3_^−^ in the water [32]. The pH depends on the partial pressure of CO_2_, dissolved matter, and temperature, characterizing and influencing the chemical and biological processes nonetheless harmful to human health, and it is controlled by the HCO_3_^−^, CO_3_^2−^, and CO_2_ equilibrium systems [31,33].

The oxidation-reduction potential (ORP) presents low values (Table 1), except for samples 5 and 12. It represents a significant indicator in the oxidative disinfection processes of the water. Disinfectants consume electrons, while contaminants with reduction characteristics donate electrons. In the case of well water, chlorine is used as a disinfectant due to the action of compounds (hypochlorous acid) released by the reaction of chloride with water [13,33]. The decrease of the ORP increases the need for chloride due to the contaminants in the water caused by reduction agents.

Sample 8 is characterized by low EC and TDS, indicating a low amount of dissolved inorganic matter in ionized form coming from surface catchments. Sample 9 has a high EC, indicating high salinity and a high amount of TDS originating from infiltrated rainwater, which dilutes the groundwater and evaporates [34]. High EC and TDS are likewise a result of anthropogenic activities. They indicate the total degree of ion concentrations and their mobility. High EC and TDS modify the taste of water [34].

The concentration of dissolved oxygen in water depends on the pressure and the temperature. Dissolved oxygen decreases in the presence of organic matter due to its oxidative degradation based on its oxygen uptake [34]. The increase in oxygen consumption is a response to water eutrophication caused by nutrient (N, P) excess. A low oxygen concentration (<5 mg/L) induces stress on the aquatic habitats and ecosystems and increases the bacteria population. The presence of fertilizers used in agricultural practices also influence the bacteria development [34].

High turbidity (sample 4) appears during strong precipitation falls and floods specific to the rainy seasons, causing siltation and sedimentation. High sedimentation and siltation are the conditions needed to increase bacteria population and metals, causing pollution [32]. The turbidity of water is caused by the presence of particulate or suspended matter which influences the penetration of light into the water [35].

Water samples are characterized by low amounts of NO_2_^−^ and NO_3_^−^, with values lower than the MACs. Sources of NO_3_^−^ and NO_2_^−^ are related to agricultural activities (such as the use of organic and chemical pesticides and fertilizers based on nitrogen and the degradation of organic waste), household activities (such as septic tanks), and industrial activities (including leaching), but they can also occur naturally (via the degradation of proteins) [36]. Health issues, namely spleen hemorrhaging or diuresis, appear if exposure to NO_2_^−^ and NO_3_^−^ occurs [34]. NO_3_^−^ is very mobile in soil and soluble in groundwater sources, and it precipitates in dry conditions as a mineral [37].

NH_4_^+^ exceeds the MAC two to seven times in all the samples. Samples 1–11 are rich in NH_4_^+^, which could lead to negative effects on human health if consumed. NH_4_^+^ can react with Cl^−^ and form chloramines [25]. Sources of NH_4_^+^ are represented by areas rich in gas and oil resources [38]. This ion is an oxidized and stable form compared to NO_3_^−^ which also originates from agricultural activities, local pedoclimatic variability, or hydrological conditions [39].

Results regarding the total hardness (H_t_) indicate a reduction of Ca and Mg (except in the soft water samples 1 and 3 and samples 8, 10, and 11). Waters with lower hardness as the MAC, or soft waters, are characterized by corrosivity and low buffering capacity [25]. Cation exchange, weathering processes of igneous rocks (including feldspar, amphibole, and pyroxene groups) and limestone, wastewaters, and industrial activities are all sources of Ca [33,37]. Mg is significant for the human body, ensuring well-functioning cells, maintaining the blood sugar level, and preventing endocrinologic, cardiologic and neurologic diseases, while a high amount of Mg could cause paralysis, nausea, and laxative effects [13,33].

Run offs or sewage discharges of fertilizers are potential sources of PO_4_^3−^ [34]. Intensive agricultural activities lead to the increase of phosphorus in water systems, favoring the excess development of algae or eutrophication [40]. Eutrophication negatively affects the quality of water (including its taste and color) and the functionality of ecosystems and biodiversity [41]. In time, the intensive use of chemical and natural fertilizers increases the amount of PO_4_^3−^ in the groundwater systems.

Samples 2 and 6 exceed two times the MAC established for Cl^−^ (250 mg/L) correlated to the highest NH_4_^+^ amounts, which is responsible for the salty taste. Those value exceedances have negative effects on agricultural crops, on human health (by affecting people with cardiovascular and kidney affections and causing laxative effects), and on household systems by corroding the plates and pipes [13,33]. The sources of Cl^−^ are represented by anthropogenic activities (including the use of fertilizers, CaCl_2_, and domestic sewers), but also by contact with soil and rocks [37]. The presence of Cl^−^ in the water systems increases the electrical conductivity and the corrosivity implicitly. In the metallic pipelines, Cl^−^ reacts with the metallic ions, forming soluble salts and increasing the metal content in the water (or the protective layer of oxide). Cl^−^ and Na^+^ are important regarding water quality because they are the most abundant electrolytes in living bodies and they play a role in acid-base balance and osmotic pressure. NaCl, MgCl_2_ and CaCl_2_ are used extensively in the chemical industry (such as for the production of NaClO, NaClO_2_, and NaOH) and for road defrosting [13].

The HCO_3_^−^ and CO_3_^2−^ sources of ions could be the natural dissolution of soil (humic acids) and rocks (including silicate minerals, limestone, and dolomite), atmospheric CO_2_, sulphate reduction processes (bacteria-organic matter), anthropogenic activities, or due to the respiration of aquatic organisms [37]. More than 50% of the samples twice exceeded the MAC established for the HCO_3_^−^ content (200 mg/L). The HCO_3_^−^ is influenced by the dissolved CO_2_, salts, cations, the pH, the temperature of the water, and other dissolved salts [21,39]. HCO_3_^−^ corelates to the hardness. Sources of high amounts of HCO_3_^−^ are related to the dissolution of soil and rocks [39]”.

The studied samples are not rich in SO_4_^2−^ (the use of fertilizer), the mineral constituents of the water, the dissolution of sulphate minerals, and the geological profile of the soil could be the sources of SO_4_^2−^) [34]. Water rich in SO_4_^2−^ could affect human health (such as by leading to cancer, heart diseases, and birth defects) [42].

The studied waters are rich in a variety of metals as shown in Table 2. The results are represented as the mean value of the samples obtained during 2021, with the standard deviation calculated using the values obtained in the 12 months of 2021. The presence of B, Ba, Li, Ga, and Sr (which are natural elements ubiquitous in the environment) is due to water-rock (including micas, granites, amphiboles, and schists) interactions [13,25]. The heavy metal content is high, and in the case of As, Fe, and Mn, it exceeds the MACs, with 1.0 µg/L for As in sample 2 and Fe in samples 5, 6, and 8. The household activities (such as leakage and waste), the industrial activities (including discharges and wastes), and the agricultural activities (including the use of herbicides, pesticides, and fertilizers) are responsible for the high metal content. Moreover, natural processes (such as water withdrawal, precipitation, and geology) could amplify the increase in metal content [43,44]. On the other hand, some microelements are essential for sustaining human health, such as Mg, Ca, K, Fe, and Zn [39].

Samples 5 and 6 are characterized by the highest Fe concentrations. If consumed, water rich in Fe can negatively affect human health. Diverse affections and diseases occur, such as cardiovascular, liver, gastric, and pulmonary issues, and rash, fatigue, and tingling [9]. The sources of Fe could be the weathering of granite or basic rocks, the chemical decomposition of ferruginous deposits, or the atmospheric exposure, which leads to Fe(II) hydrolysis in the presence of dissolved oxygen and generates Fe(OH)_3_ [25,38]. Fe is a nutrient significant for aquatic organisms as well, but a high amount could cause negative effects on human health, such as breathing problems, tingling, and rash [9]. The release of Fe is influenced by the variation of pH, dissolved oxygen, alkalinity, organic matter, and micro-organisms [34].

A metallic, unpleasant taste and mud odor characterize Mn-rich water (samples 5, 6, and 8), which may cause apathy, muscular pain, and anorexia [1,8]. Sources of Mn are represented by industrial activities (such as the production of alkaline batteries or cleaning products), agricultural activities (including the use of fungicides and fertilizers), or mining activities [45]. Nevertheless, Mn is also an abundant element naturally found in the crust of Earth [45]. The presence of Mn in the water distribution system forms deposits that could slough off as a black precipitate. The nervous system is affected by the ingestion of food and water contaminated with Mn (it may lead to Parkinson’s disease and altered cognitive and motor functions) [45].

The high values of Na (sample 6) could be caused by the dissolution of soil salts and rock (forming minerals), septic tank infiltrations, and cation exchange interactions between the clay fraction and groundwater, suggesting significant water-rock interactions [21,46]. According to Petrovic [38], water with a considerable amount of Na is characterized by rich mineralization processes implying a high number of trace-elements. A high Na concentration causes heart, renal, and neurologic diseases [47]. Individuals with renal and cardiovascular affections need water with little Na [47]. The geological structure (alkali feldspar), the processes of ionic exchange (the adsorption of Ca from the rock and the enrichment of water with Na), the processes of alienation of aluminosilicate minerals of sodium, and the active weathering processes are responsible for the presence of Na in water samples [13,33].

Samples 7–10 are characterized by high amounts of K, exceeding the MAC two to four times and having the use of chemical and organic manure or human waste as potential sources [33,39]. High amounts of K in water are related to the use of fertilizers rich in K in agricultural practices [46].

Water rich in Al (5), if ingested, could cause chromosome aberrations in barley meri-stem cells. However, a low amount of Al in water poses negative human health effects (non-carcinogenic) [1]. A possible source is the use of Al_2_(SO_4_)_3_ in the water treatment process.

Samples 1, 5, and 9 are rich in Ni. The pH, soil, and depth influence the amount of Ni. The Ni amounts that are higher than the background value are related to mining plants and industrial waste. Given the carcinogenic characteristics of heavy metals, Ni combined with Cd, Cr, and As alters and damages the DNA [9].

The highest value of As is attributed to sample 2, which exceeds the MAC, while sample 1 slightly reaches the MAC. After ingestion, As is rapidly absorbed from the gastrointestinal tract and further metabolized [48]. High amounts of As negatively affect human health by causing vascular and skin diseases, vomiting, diarrhea, encephalopathy, and cancer [48]. Due to the geochemical conditions, As present in groundwater is vulnerable to sharp fluctuations [49].

The relatively high amounts of Pb in the studied samples (1, 3, 4, 12) are potentially caused by the improper discharges of industrial activities loaded directly into the groundwater sources, agricultural practices (including fertilizers and pesticides), or natural processes related to the weathering of minerals (such as dolomite, marble, and limestone) [9,50]. Negative health effects could appear in the liver, thyroid, and bones, and they could also lead to high blood pressure, brain damage, infertility, and even cancer [9,50].

The presence of Cd in the studied samples, especially in sample 4, is attributed to natural and anthropogenic sources [12,50]. A source of drinking water that is high in Cd could cause immediate poisoning and diarrhea, damaging the kidney and liver [1,9].

Generally, Cu (sample 8 slightly reaches the MAC) occurs due to natural processes (like rock degradation) and anthropogenic activities (such as mining, municipal, industry, and agriculture activities) as well [1]. Stomach-ache, cerebral pain, and irritated eyes and nose occur if a water rich in Cu is consumed [9].

Sample 1 is also rich in Cr and slightly reaches the MAC, probably due to the presence of magnesiochromite, which are mafic and ultramafic rocks of chromite in which, through weathering processes, Cr ions are released into the water systems [1,50]. According to Ali [12], Cr is a powerful oxidizing agent, and it is entirely adsorbed by aquatic vegetation, indicating direct intake from sediments.

Zn is also a natural element. The interaction of groundwater with the surrounding rocks slowly enriches the water body through delayed exchange (as in the case of sample 1- Table 2) [51]. The content of inorganic carbon and the pH influence the solubility of Zn [25]. Zn is characterized by high mobility in the water systems, induces opalescence, has an astringent taste, and is released into the environment due to worn rubber tires of vehicles and coal combustion [1]. Zn is essential for living creatures, although it is toxic in high concentrations, causing cardiovascular issues, affecting immunity, causing cell mutations, increasing the permeability of the cell membrane, and causing death [9,52].

The release of heavy metals in the study area is related to geological conditions, namely the presence of volcanic rocks (including andesite and rocks rich in sulphide veins) and natural processes implying rocks and minerals (such as degradation, weathering, and oxidation) [50].

### 3.2. Water Typology

#### 3.2.1. Piper and Gibbs Diagrams

**Figure 2:**
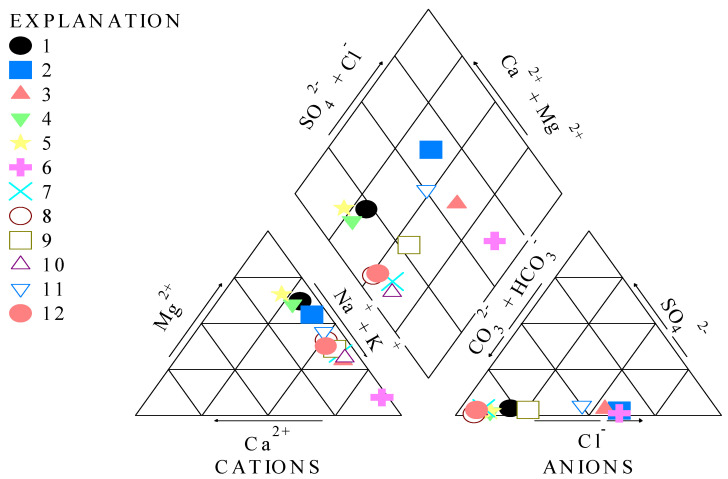
Piper trilinear diagram for the water samples (1–12).

A Piper diagram was plotted for all 12 water samples with the help of concentrations of four major cations (Ca^2+^, Mg^2+^, Na^+^ and K^+^), four anions (Cl^−^, SO_4_^2−^, CO_3_^2−^ and HCO_3_^−^), and the TDS. According to the plot and Manoj [53] classification, the studied samples are classified into mixed Ca^2+^-Mg^2+^-Cl^−^ type (sample 2), Na^+^-Cl^−^ type (samples 3 and 6), Ca^2+^-HCO_3_^−^ type (samples 1, 5, and 4), Na^+^-HCO_3_^−^ type (samples 7, 8, 10, and 12), and mixed Ca^2+^-Na^+^-HCO_3_^−^ type (sample 9). Thus, sample 11 has a mixed typology according to the diamond plot. There is no dominant type according to the anion triangle, and there is an Na^+^-K^+^ type according to the cation triangle (Figure 2). The presence of silicate, igneous rocks, minerals, and weathering contributes to the dominance of waters of type Ca^2+^-Na^+^-HCO_3_^−^. The samples with the Na^+^-HCO_3_^−^ typology are characterized by the presence of reverse ionic exchange processes of Ca^2+^ and Na^+^ and the weathering of albite or other igneous rock minerals [37]. According to Rupias [37], minerals containing Ca^2+^ and Na^+^ are susceptible to the weathering processes.

**Figure 3:**
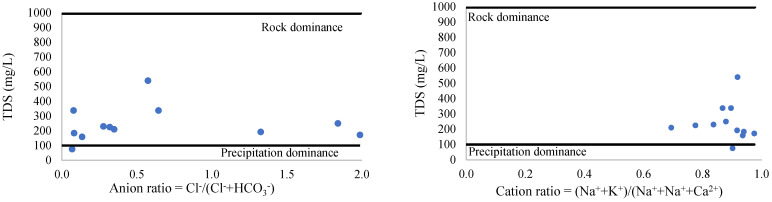
Gibbs diagrams indicating anion and cation ratio for all water samples (1–12).

The Gibbs diagram indicates three distinct fields, namely evaporation, precipitation, and rock–water interaction dominance areas [37]. According to Figure 3, the majority of studied samples fall into the rock–water interaction dominance, indicating that the water samples originate from the interaction of the chemistry of percolated water under the lakes and rock chemistry.

According to Gibbs [18] and Shah [21], a Gibbs diagram indicates the natural mechanisms controlling water systems, such as evaporation, rock, or precipitation dominance. Gibbs plots are based on different physico-chemical parameters (anions and cations) related to the TDS. In the present study, two Gibbs diagrams were applied to all 12 water samples based on the anion ratio (Cl^−^/(Cl^−^ + HCO_3_^−^) and the cation ratio ((Na^+^ + K^+^)/(Na^+^ + K^+^ + Ca^2+^)) (Figure 3). According to the Gibbs plots, generally, the studied water samples are characterized by rock dominance or weathering dominance. The Gibbs ratio ranges from 0.07 to 1.99 in the case of the anion ratio, while the Gibbs ratio related to the cation content ranges between 0.69 and 0.98. This indicates that weathering is the possible source of the hydrochemistry of the studied water samples.

#### 3.2.2. Stiff and Schoeller Diagrams

**Figure 4:**
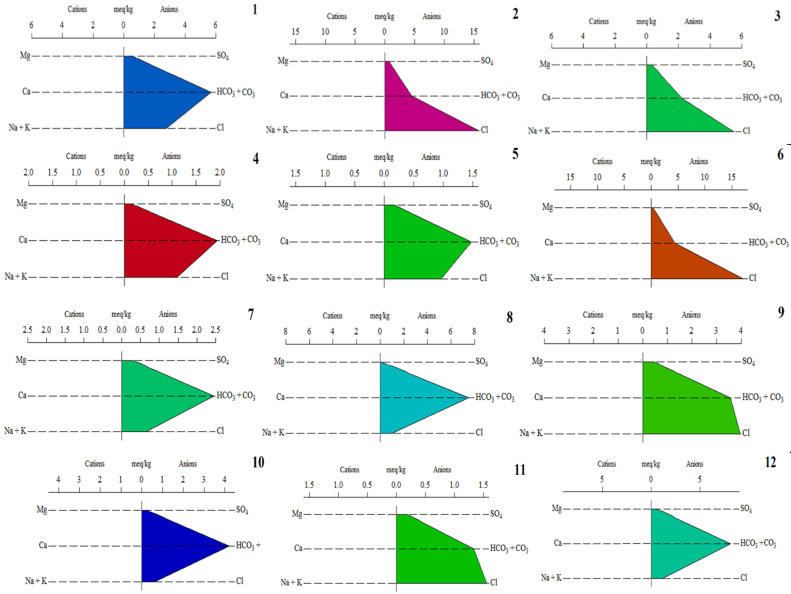
Stiff diagrams for all water samples (1–12).

A Stiff diagram (Figure 4) is a graphical representation of the major ions identified and determined from the water samples. The used ions are Mg^2+^, Ca^2+^, Na^+^, K*, SO_4_^2−^, Cl^−^, HCO_3_^−^, and CO_3_^2−^. Characteristically, anions are placed on the right side of the center axis, while the cations are placed on the left side. This way, equivalent amounts are presented. The amounts are indicated in meq/L (milliequivalents/L). According to the Stiff plot, the dominant types of water are represented by HCO_3_^−^ + CO_3_^2−^ and Cl^-^ (Figure 4).

**Figure 5:**
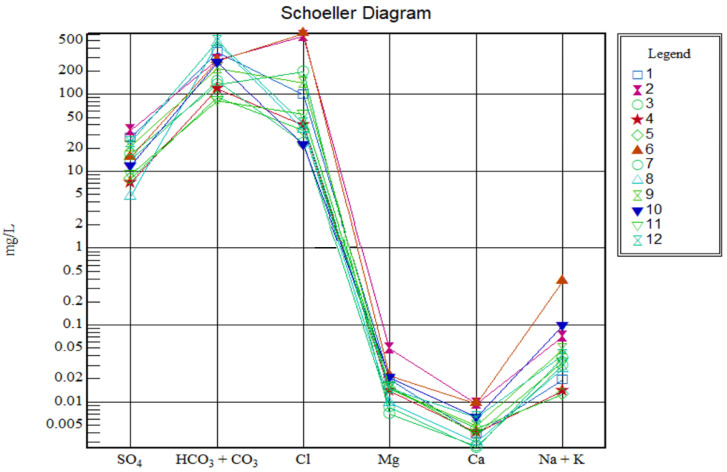
Schoeller diagrams for all water samples (1–12).

According to the Stiff and Schoeller plots (Figure 4 and Figure 5), the cation content is not notable compared to the anion content. More than 50% of the samples are dominated by the HCO_3_^−^ + CO_3_^2−^ content (samples 1, 4, 5, 7, 8, 10, and 12). Less than 50% of the samples are characterized by high amounts of Cl^−^ (samples 2, 3, 6, and 11). Sample 9 is dominated by Cl^−^ and HCO_3_^−^ + CO_3_^2−^. The same trend and results, expressed in mg/L, are shown with the help of the Schoeller plot (Figure 5), and show low amounts of cations and the dominance of HCO_3_^−^ + CO_3_^2−^ and Cl^−^ anions.

#### 3.2.3. Correlations between the Metal Content and the Pollution Indices

Pearson’s correlation was determined between the metal content (As, Al, Cd, Cr, Cu, Mn, Ni, Zn, Fe) and the *PI* and *HEI* scores. As indicated in Table 3, a positive correlation is observed between As-Fe, Fe-Mn, Mn-Ni, and Cr-Zn. Also, significant correlations are established between the metal concentrations and the pollution indices, such as Fe-PI, Fe-*HEI*, As-*PI*, Al*-HEI*, and Mn-*HE*I. The highest *PI* score correlates with the highest As amount, followed by *PI* correlated with the highest Fe and Mn concentrations.

### 3.3. Pollution Indices

**Figure 6:**
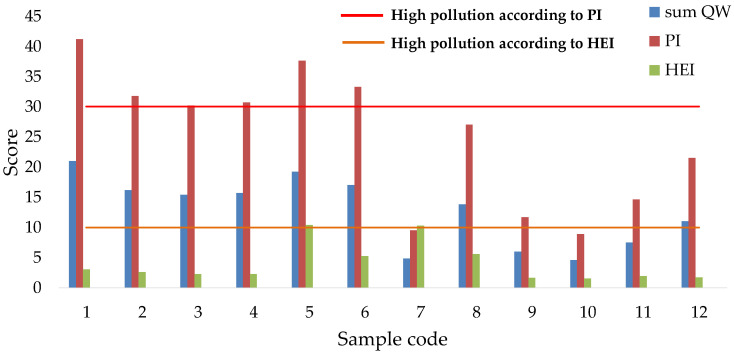
*PI* and *HEI* scores and the corresponding pollution level of the studied water samples.

The results regarding the pollution status based on the *PI* and *HEI* results indicate three different pollution levels. According to the *PI* scores, samples 1–6 are characterized by a high pollution level, while samples 7, 9–12 have a low pollution level, and samples 8 and 12 are characterized by a medium level of pollution, as indicated in Figure 6. The mean value is 24.8, while the lowest value is 8.90 (sample 10), followed by 7 < 9 < 11 < 12 < 8 < 3 < 4 < 2 < 6 < 5 < 1. Sample 1 is characterized by the highest score due to the highest concentrations of Al, Pb, and Cr obtained for all samples.

Based on the *HEI* results, sample 5 is characterized by a medium pollution level, while the rest of the samples have a low level of metal pollution. Generally, as indicated in Figure 6, the medium value is 4.05, indicating a low level of pollution. The highest value is 10.4, obtained for sample 5, followed by 7 > 8 > 6 > 1 > 2 > 3 > 4 > 11 > 12 > 9 > 10. The highest score is directly proportional to the highest Fe and Mn concentrations. Certain scores exceed the MACs. Other studies in different parts of the world used pollution index methods in order to determine the pollution level of water. In Guanzhong Plain, China, *HPI* and *HEI* scores ranged from 0.33–28.5 and 0.06–4.57, indicating a low level of metal pollution [29]. In Angul, India, waters were characterized by a low pollution level, as reflected by the *HPI* scores (30–87) [54].

In this study’s location (Maramures, the north-western part of Romania), *HPI* results range between 5.5–97.7, indicating no metal pollution in the water samples used as drinking water. *HEI* scores range from 1.5–14.0, indicating no pollution with metals [55]. Alluvial aquifers situated in the Maramures Depression are studied in the frame of heavy metal pollution assessment with the help of pollution indices (*HEI* and *HPI*). The results indicate three types of pollution statuses: low, medium, and high. The *HPI* and the *HEI* range from 5.6 to 234 and 0.4 to 59, respectively. The high scores are attributed to the high amounts of Mn and Fe caused by the water–rock interactions and the presence of organic colloids and humid materials [56].

On the other hand, in the south-eastern part of the country (Dobrogea), studied waters are characterized by two classes of samples, including unpolluted and polluted with the studied metals [14]. *HPI* results range between 89.2 and 196 due to the high amounts of Cr, which exceed the MAC. *HEI* scores range from 0.1 to 1.0, indicating two pollution statutes [14].

### 3.4. Health Risk Assessment

**Figure 7:**
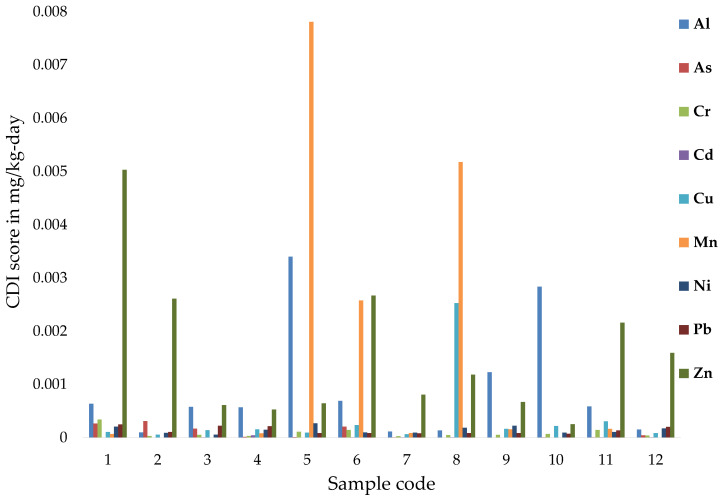
CDI scores obtained for the studied water samples if consumed.

The content of the metals is studied concerning the human health risk assessment. The risk assessment is based on the oral intake of water in the case of adults. The results regarding the chronic daily intake (*CDI*) and the hazard quotient (*HQ*) are indicated in Figure 7 and Table 4.

The CDI values are indicated in Figure 7. Based on the obtained results, the most relevant chronic daily intake for the studied metals through water intake is represented by Mn, followed by Zn < Al < Cu < Cr < As < Ni < Pb < Cd. Generally, the scores reveal that the highest CDI values are obtained in the cases of samples 5, 8, and 1. The highest metal consumed and absorbed through water ingestion is Zn with a mean value of 1.6 × 10^−3^ mg/kg-day (Figure 7). The Zn concentration is increased due to the interactions between water–rock. A higher concentration could affect the water quality and, if consumed, human health, causing cardiovascular disease and death [9,52]. Mostly, Mn and Al are also absorbed in a high amount, with mean values of 1.3 × 10^−3^ mg/kg-day Mn and 0.9 × 10^−3^ mg/kg-day Al, which could be a major health risk if ingested, affecting the neurologic system and cells [1,45].

When calculating HQ and HI (which are conservative health risk assessment tools), the non-carcinogenic risk related to toxic element exposure (Al, As, Cr, Cd, Cu, Mn, Ni, Pb, and Zn) is estimated related to the ingestion of water (oral toxicity) in the case of adults. HQ scores depend on the body weight, the volume of water consumed by the inhabitant, the exposure frequency, and the duration. Table 4 indicates the mean scores obtained for HQ. Generally, overall *HQ* results are lower than 1.0, indicating that if consumed, the drinking water samples present no non-carcinogenic risk associated with human health, except for samples 5 and 10, which are characterized by high amounts of Al. Consequently, Al contributes most to the exposure of non-cancer risk. Sources of Al are related to the chemical processes applied in the water treatment. Cells can be affected by the presence of Al in ingested water [1]. Mn and Zn follow Al as the main contributors to ingestion exposure and its human health impact. The *HQ* scores were all negative, ranging from 2.0 × 10^−5^ to 8.4 × 10^−2^, except for two samples characterized by high amounts of Al. The highest *HQ* value is obtained in samples 5 and 10, whose values are 2.4 and 2.0, respectively, while the lowest values are obtained in samples 7, 2, and 12. Mostly, the result of the present study indicates that a chance of pollution with the studied metals can occur which affects human health through the ingestion pathway. The hazard quotient caused by metal intake through water ingestion indicates a leading approach comparable to different studies that helps estimate health risks and protects the population. Similarly, *HQ* is applied in different parts of the country in order to assess the risk of ingestion metals through water. Studies in the south-eastern part of the country indicate *HQ* scores lower than the critical value, varying between 2.6 × 10^−2^ and 2.8 × 10^−2^ for *HQ*_Cd_. *HQ*_Cr_ varies between 3.5 × 10^−2^ and 4.3 × 10^−1^, *HQ*_Cu_ varies between 9.5 × 10^−4^ and 1.3 × 10^−3^, *HQ*_Ni_ varies between 2.4 × 10^−3^ and 8.2 × 10^−3^, *HQ*_Pb_ varies between 2.1 × 10^−5^ and 2.3 × 10^−5^, and *HQ*_Zn_ varies between 1.2 × 10^−4^ and 4.1 × 10^−4^ [14].

*HI* was calculated by accumulating the *HQ* for each studied metal. *HI* results indicate no potential risk related to ingesting the studied waters for the majority of the studied samples, except for samples 5 and 10. *HI* scores range between 0.03 and 2.5 for samples 5 and 10, and the lowest value is obtained in the case of sample 7, followed by 2 < 12 < 8 < 11 < 3 < 4 < 6 < 1 < 9.

## 4. Conclusions

According to the obtained results, the studied groundwater samples collected from the protected Tisa River Basin are characterized by high amounts of Cl^−^, NH_4_^+^, Fe, and Mn, exceeding the MACs. The samples are also rich in Pb, Cu, and Mg, but with amounts lower than the MACs. The Piper diagram indicates that the studied water samples are generally classified into five types of water (Na^+^-Cl^−^, Na^+^-HCO_3_^−^, Ca^2+^-HCO_3_^−^, mixed Ca^2+^-Mg^2+^-Cl^−^, and Ca^2+^-Na^+^-HCO_3_^−^). According to the Gibbs plot, all water samples are characterized by a weathering or rock dominance. Based on the Pearson correlation, a positive correlation is noticed between Cr-Zn, As-Fe, Fe-Mn, and Mn-Ni, indicating the same pollution source. A positive correlation is observed between the highest metal content and the score for the pollution indices (*PI* and *HEI*). Based on the two metal pollution indices results, three different pollution levels are determined. The risk assessment analysis indicates that there are no non-carcinogenic risks related to the studied metals determined in water samples, except for two samples, which are characterized by high amounts of Al. Consequently, it is recommended that the studied water samples should be further monitored and treated if they are used for drinking purpose. Due to its approach, this study is significant for future research related to determining and assessing the quality of water sources situated in areas where agricultural practices are implemented. This way, the population is informed and aware, and possible negative effects on health related to the ingestion of poor-quality water will be prevented. Sustainable policies and protection policies need to be framed in order to decrease the possible negative effects on human health. This study’s results could be used for management mitigation efforts regarding poor-quality water sources and in medicine research. Perspectives for new research relate to the identification of diseases and their negative or positive effects on organs.

## Figures and Tables

**Table 1:** Quality index parameters (mean ± standard deviation) of the groundwater samples (1–12).

Sample	1	2	3	4	5	6	7	8	9	10	11	12	MAC *
**pH**	7.94 ± 0.45	7.35 ± 0.28	7.42 ± 0.26	7.82 ± 0.39	7.43 ± 0.35	7.23 ± 0.21	7.19 ± 0.18	7.27 ± 0.23	7.25 ± 0.21	7.70 ± 0.34	7.65 ± 0.33	7.52 ± 0.31	6.5–9.5
**ORP [mV]**	157 ± 22	191 ± 29	51.1 ± 10	202 ± 31	233 ± 35	219 ± 33	215 ± 32	195 ± 30	175 ± 26	178 ± 27	279 ± 25	100 ± 16	-
**EC [μS/cm]**	360 ± 35	390 ± 38	300 ± 29	351 ± 33	329 ± 36	269 ± 29	249 ± 31	118 ± 17	844 ± 92	287 ± 22	528 ± 48	528 ± 56	2500
**DO [mg/L]**	6.99 ± 3.21	4.79 ± 2.82	6.10 ± 3.16	7.76 ± 2.85	3.70 ± 1.86	5.62 ± 2.45	5.71 ± 2.38	6.63 ± 2.95	7.51 ± 2.46	10.3 ± 4.2	8.15 ± 2.58	9.93 ± 3.34	-
**SO [%]**	50.9 ± 3.5	40.1 ± 3.1	49.6 ± 3.5	62.7 ± 3.0	30.4 ± 2.1	45.8 ± 2.6	54.4 ± 2.6	57.8 ± 3.2	63.7 ± 2.7	84.7 ± 3.9	60.3 ± 2.64	83.1 ± 3.8	-
**T (NTU)**	0.36 ± 0.14	1.07 ± 0.11	2.75 ± 0.65	**10.70 ± 1.12**	2.71 ± 0.75	2.16 ± 0.36	0.48 ± 0.16	0.77 ± 0.24	0.53 ± 0.16	0.80 ± 0.24	1.85 ± 0.64	0.63 ± 0.16	<5
**NH_4_^+^ [mg/L]**	**2.18 ± 0.35**	**3.71 ± 0.72**	**0.94 ± 0.22**	**0.59 ± 0.15**	**0.85 ± 0.22**	**2.79 ± 1.12**	**1.65 ± 0.36**	**1.68 ± 0.28**	**2.45 ± 0.47**	**2.10 ± 0.42**	**0.74 ± 0.13**	0.50 ± 0.08	0.5
**NO_3_^−^ [mg/L]**	0.89 ± 0.13	0.75 ± 0.11	1.09 ± 0.23	0.32 ± 0.06	1.24 ± 0.26	1.75 ± 0.32	1.24 ± 0.27	5.78 ± 1.12	6.50 ± 1.21	2.68 ± 0.56	15.4 ± 1.76	2.84 ± 0.46	50
**NO_2_^−^ [mg/L]**	0.002 ± 0.001	0.002 ± 0.001	0.015 ± 0.002	0.003 ± 0.001	0.002 ± 0.001	0.002 ± 0.001	0.002 ± 0.001	0.006 ± 0.001	0.002 ± 0.001	0.006 ± 0.001	0.025 ± 0.004	0.003 ± 0.001	0.5
**H_t_ [^o^G]**	**3.86 ± 0.42**	16.8 ± 0.2	**3.20 ± 0.07**	11.3 ± 0.1	8.74 ± 0.09	19.3 ± 0.3	7.56 ± 0.11	5.90 ± 0.33	11.4 ± 0.1	5.71 ± 0.28	5.40 ± 0.29	8.70 ± 0.14	>5
**Cl^−^ [mg/L]**	98 ± 6	**561 ± 43**	194 ± 12	39 ± 6	34 ± 5	**606 ± 56**	23 ± 3	35 ± 5	140 ± 15	22 ± 3	55 ± 8	41 ± 5	250
**PO_4_^3−^ [mg/L**	0.05 ± 0.01	0.07 ± 0.02	0.02 ± 0.01	0.02 ± 0.01	0.02 ± 0.01	0.05 ± 0.01	0.02 ± 0.01	0.02 ± 0.01	0.12 ± 0.03	0.25 ± 0.04	0.02 ± 0.01	0.22 ± 0.05	0.5
**HCO_3_^−^ [mg/L]**	**354 ± 16**	**305 ± 13**	146 ± 11	122 ± 12	97.6 ± 7.5	**305 ± 16**	171 ± 15	**512 ± 23**	**244 ± 19**	**268 ± 32**	85.4 ± 0.8	**525 ± 27**	200
**CO_3_^2−^ [mg/L]**	174 ± 15	144 ± 11	56.0 ± 1.6	173 ± 19	155 ± 13	172 ± 19	110 ± 13	178 ± 18	207 ± 22	109 ± 15	10.0 ± 1.7	221 ± 24	-
**SO_4_^2−^ [mg/L]**	26.6 ± 1.8	34.1 ± 2.6	16.4 ± 1.1	7.00 ± 0.46	7.90 ± 0.23	15.0 ± 1.7	14.0 ± 1.9	4.67 ± 0.22	20.6 ± 2.3	11.7 ± 3.1	8.90 ± 1.9	23.8 ± 2.6	250
**TDS [mg/L]**	230 ± 20	250 ± 23	192 ± 18	225 ± 21	210 ± 29	172 ± 19	159 ± 14	75.5 ± 8.9	540 ± 43	184 ± 19	338 ± 28	338 ± 35	-

* according to Law 311 (2004) and WHO (2011), regarding the drinking water quality. The values in bold exceed the MACs related to Law 311 from 2004.

**Table 2:** The metals and heavy metals characteristics (mean ± standard deviation) of the studied water samples.

Sample [μg/L]	1	2	3	4	5	6	7	8	9	10	11	12	MAC *
**Ag**	0.02 ± 0.01	0.02 ± 0.01	5.04 ± 1.04	0.05 ± 0.01	0.02 ± 0.01	0.02 ± 0.01	0.02 ± 0.01	0.41 ± 0.01	0.02 ± 0.01	0.02 ± 0.01	0.02 ± 0.01	0.02 ± 0.01	**-**
**Al**	22.3 ± 3.1	3.45 ± 0.38	20.3 ± 2.8	19.9 ± 1.5	119 ± 11	24.2 ± 1.4	4.04 ± 0.18	4.80 ± 0.25	43.2 ± 3.8	99.3 ± 6.5	20.6 ± 1.3	5.27 ± 0.62	**200**
**As**	9.23 ± 2.62	**10.9 ± 3.4**	5.93 ± 2.1	0.43 ± 0.13	0.39 ± 0.10	7.26 ± 3.13	0.19 ± 0.06	0.02 ± 0.01	0.03 ± 0.01	0.25 ± 0.06	0.13 ± 0.02	1.54 ± 0.08	**10**
**Au**	0.02 ± 0.01	0.34 ± 0.05	0.02 ± 0.01	0.02 ± 0.01	0.02 ± 0.01	0.16 ± 0.03	0.06 ± 0.01	0.02 ± 0.01	0.02 ± 0.01	0.02 ± 0.01	0.02 ± 0.01	0.03 ± 0.01	**-**
**Ba**	22.4 ± 2.5	75.4 ± 11.1	28.5 ± 3.3	24.7 ± 2.6	28.1 ± 2.7	91.2 ± 14.3	25.3 ± 2.1	89.0 ± 15.2	92.7 ± 20.1	109 ± 25.3	22.9 ± 2.2	41.7 ± 8.3	**700**
**Bi**	0.02 ± 0.01	0.10 ± 0.02	0.14 ± 0.03	0.02 ± 0.01	0.02 ± 0.01	0.02 ± 0.01	0.02 ± 0.01	0.02 ± 0.01	0.02 ± 0.01	0.02 ± 0.01	0.02 ± 0.01	0.02 ± 0.01	**-**
**Ca**	3760 ± 355	9520 ± 768	2670 ± 249	3950 ± 436	4490 ± 598	9430 ± 875	2540 ± 206	2990 ± 309	4890 ± 572	6310 ± 655	3830 ± 394	6220 ± 786	**100,000**
**Cd**	0.10 ± 0.03	0.10 ± 0.02	0.17 ± 0.04	1.47 ± 0.37	0.09 ± 0.02	0.25 ± 0.08	0.13 ± 0.03	0.18 ± 0.04	0.07 ± 0.02	0.08 ± 0.03	0.15 ± 0.03	0.15 ± 0.04	**5**
**Co**	0.24 ± 0.09	0.07 ± 0.02	0.10 ± 0.02	0.20 ± 0.05	0.36 ± 0.13	0.15 ± 0.06	0.07 ± 0.01	0.16 ± 0.04	0.13 ± 0.03	0.24 ± 0.04	0.25 ± 0.05	0.64 ± 0.12	**-**
**Cr**	11.9 ± 1.7	1.07 ± 0.13	1.81 ± 0.08	1.05 ± 0.18	3.88 ± 0.45	5.07 ± 0.62	0.91 ± 0.18	1.72 ± 0.21	1.83 ± 0.42	2.37 ± 0.58	4.98 ± 0.46	1.37 ± 0.19	**50**
**Cs**	0.04 ± 0.01	0.07 ± 0.02	0.06 ± 0.02	0.03 ± 0.01	0.13 ± 0.03	0.07 ± 0.02	0.06 ± 0.03	0.21 ± 0.08	0.02 ± 0.01	0.02 ± 0.01	0.03 ± 0.01	0.07 ± 0.02	**-**
**Cu**	3.74 ± 1.05	1.88 ± 0.76	4.87 ± 1.34	5.45 ± 1.82	3.25 ± 1.02	8.24 ± 2.31	2.21 ± 0.64	88.4 ± 11.1	5.83 ± 0.01	7.71 ± 1.04	10.8 ± 1.49	3.01 ± 0.51	**100**
**Fe**	80 ± 18	180 ± 20	120 ± 35	140 ± 51	**680 ± 84**	**380 ± 63**	80 ± 21	70 ± 17	110 ± 42	90 ± 23	160 ± 37	80 ± 15	**200**
**Ga**	8.95 ± 0.75	29.5 ± 3.14	13.0 ± 0.27	10.1 ± 0.28	11.2 ± 0.35	36.1 ± 4.8	10.7 ± 1.1	38.6 ± 4.6	38.8 ± 3.9	45.4 ± 5.2	9.24 ± 1.07	17.0 ± 1.78	**-**
**Ge**	0.02 ± 0.01	0.02 ± 0.01	0.02 ± 0.01	0.05 ± 0.02	0.05 ± 0.01	0.04 ± 0.02	0.04 ± 0.03	0.02 ± 0.01	0.02 ± 0.01	0.02 ± 0.01	0.04 ± 0.02	0.04 ± 0.01	**-**
**Hf**	0.18 ± 0.04	0.02 ± 0.01	0.02 ± 0.01	0.10 ± 0.02	0.02 ± 0.01	2.39 ± 0.28	0.02 ± 0.01	0.02 ± 0.01	0.02 ± 0.01	0.02 ± 0.01	0.02 ± 0.01	17.6 ± 2.8	**-**
**Ir**	0.02 ± 0.01	0.02 ± 0.01	0.02 ± 0.01	0.11 ± 0.03	0.02 ± 0.01	0.27 ± 0.08	0.02 ± 0.01	0.03 ± 0.01	0.02 ± 0.01	0.02 ± 0.01	0.02 ± 0.01	0.03 ± 0.02	**-**
**K**	1930 ± 548	7440 ± 231	2660 ± 107	3180 ± 362	1370 ± 472	6110 ± 779	**19,860 ± 2077**	**11,020 ± 1085**	**28,350 ± 2947**	**45,410 ± 4625**	4200 ± 451	4460 ± 473	**10,000**
**Li**	2.71 ± 1.12	5.40 ± 1.78	2.00 ± 0.47	4.41 ± 0.84	9.14 ± 2.39	11.2 ± 3.08	2.85 ± 0.75	4.21 ± 1.64	4.70 ± 1.89	7.84 ± 2.27	1.73 ± 0.47	4.34 ± 1.39	**30**
**Mg**	19,720 ± 1645	48,860 ± 2185	7010 ± 545	13,660 ± 1245	15,050 ± 1657	21,570 ± 2378	8520 ± 795	9620 ± 1388	14,570 ± 1672	20,820 ± 2527	15,740 ± 1945	14,600 ± 2385	**50,000**
**Mn**	2.46 ± 0.74	0.02 ± 0.01	0.02 ± 0.01	2.88 ± 1.03	**273 ± 29**	**90.1 ± 12.4**	3.04 ± 0.54	**181 ± 20**	5.47 ± 1.12	0.02 ± 0.01	5.71 ± 1.38	0.02 ± 0.01	**50**
**Mo**	6.65 ± 1.04	2.69 ± 0.58	0.23 ± 0.04	0.47 ± 0.08	1.11 ± 0.37	0.91 ± 0.25	0.61 ± 0.18	0.19 ± 0.04	0.37 ± 0.12	0.24 ± 0.13	0.27 ± 0.11	0.64 ± 0.22	**-**
**Na**	17,380 ± 2544	61,790 ± 7245	26,820 ± 3127	10,480 ± 1783	8800 ± 947	**363,940 ± 71,253**	17,300 ± 7582	16,380 ± 1752	26,870 ± 3038	52,810 ± 5461	28,950 ± 3017	36,410 ± 3816	**200,000**
**Nb**	0.02 ± 0.01	0.05 ± 0.02	0.05 ± 0.03	0.04 ± 0.02	0.09 ± 0.02	0.03 ± 0.01	0.02 ± 0.01	0.02 ± 0.01	0.02 ± 0.01	0.02 ± 0.01	0.02 ± 0.01	0.07 ± 0.03	**-**
**Ni**	7.29 ± 2.16	3.16 ± 0.71	1.89 ± 0.32	5.14 ± 1.42	9.45 ± 2.38	3.44 ± 1.07	3.27 ± 1.12	6.42 ± 2.09	7.90 ± 3.15	3.21 ± 1.06	3.65 ± 0.89	5.98 ± 1.27	**20**
**Pb**	8.72 ± 1.32	3.64 ± 0.42	7.89 ± 1.78	7.50 ± 1.62	2.96 ± 0.31	3.00 ± 0.33	2.90 ± 0.49	3.08 ± 0.27	2.94 ± 0.37	2.51 ± 0.39	4.83 ± 0.55	7.04 ± 1.34	**10**
**Rb**	2.16 ± 0.22	3.24 ± 0.35	1.28 ± 0.14	0.52 ± 0.12	1.67 ± 0.46	0.49 ± 0.18	4.17 ± 0.77	1.70 ± 0.57	2.35 ± 0.72	1.52 ± 0.16	2.19 ± 0.24	0.65 ± 0.19	**-**
**Sb**	3.36 ± 0.47	1.73 ± 0.22	1.09 ± 0.17	1.37 ± 0.23	1.34 ± 0.34	**13.3 ± 2.3**	0.25 ± 0.09	0.13 ± 0.03	1.12 ± 0.18	1.77 ± 0.23	0.16 ± 0.05	0.68 ± 0.29	**5**
**Sn**	854 ± 127	79.2 ± 19.7	64.3 ± 15.1	0.09 ± 0.02	0.02 ± 0.01	10.1 ± 0.17	176 ± 21	0.40 ± 0.12	1.51 ± 0.32	0.02 ± 0.01	4.42 ± 0.54	50.3 ± 11.1	**-**
**Sr**	140 ± 20	530 ± 61	120 ± 17	210 ± 34	200 ± 81	950 ± 103	160 ± 34	190 ± 42	350 ± 25	530 ± 77	250 ± 37	310 ± 34	**7000**
**Ti**	21.0 ± 3.1	50.9 ± 7.4	24.6 ± 3.6	25.1 ± 3.2	43.3 ± 5.5	40.0 ± 3.9	14.5 ± 1.8	15.8 ± 2.2	25.2 ± 4.5	32.7 ± 3.8	20.3 ± 2.4	32.9 ± 4.6	**-**
**Tl**	0.47 ± 0.12	0.09 ± 0.03	0.23 ± 0.07	0.18 ± 0.05	0.08 ± 0.04	0.14 ± 0.05	0.14 ± 0.04	0.27 ± 0.13	0.04 ± 0.01	0.05 ± 0.02	0.03 ± 0.01	0.37 ± 0.16	**-**
**V**	0.36 ± 0.14	0.37 ± 0.11	0.42 ± 0.16	0.25 ± 0.08	1.29 ± 0.33	0.32 ± 0.06	0.23 ± 0.04	0.04 ± 0.01	0.11 ± 0.03	0.17 ± 0.05	0.92 ± 0.29	0.22 ± 0.05	**-**
**Zn**	176 ± 20	91.4 ± 8.8	21.4 ± 2.6	18.4 ± 2.1	22.5 ± 3.1	93.4 ± 12.4	28.2 ± 3.6	41.5 ± 4.8	23.4 ± 2.5	8.75 ± 1.22	75.6 ± 12.5	55.7 ± 6.8	**5000**
**Zr**	4.71 ± 0.56	13.49 ± 1.52	0.56 ± 0.18	0.31 ± 0.09	0.55 ± 0.08	1.27 ± 0.46	0.06 ± 0.02	0.06 ± 0.01	0.10 ± 0.03	0.12 ± 0.04	0.51 ± 0.11	9.45 ± 1.57	**-**

* according to Law 311 (2004) and WHO (2011), related to the drinking water quality. The values in bold exceed the MACs related to Law 311 from 2004.

**Table 3:** Pearson’s correlation coefficient between the heavy metals and the PI, HEI scores.

Variables	Fe	As	Al	Cd	Cr	Cu	Mn	Ni	Pb	Zn	*PI*	*HEI*
**Fe**	**1**	0.023	0.614 *	−0.073	0.124	−0.198	**0.753**	0.389	−0.319	−0.068	**0.501**	**0.888**
**As**		**1**	−0.309	−0.182	0.424	−0.260	−0.227	−0.273	0.316	**0.720**	**0.596**	−0.087
**Al**			**1**	−0.162	0.105	−0.210	0.474	0.388	−0.357	−0.347	0.052	**0.682**
**Cd**				**1**	−0.216	−0.046	−0.137	−0.043	0.374	−0.223	0.175	−0.130
**Cr**					**1**	−0.125	0.039	0.289	0.361	**0.816**	0.479	0.191
**Cu**						**1**	0.457	0.155	−0.240	−0.091	0.013	0.170
**Mn**							**1**	0.565	−0.408	−0.160	0.437	**0.931**
**Ni**								**1**	−0.029	0.078	0.313	**0.572**
**Pb**									**1**	0.371	0.416	−0.322
**Zn**										**1**	0.491	−0.085
***PI***											**1**	**0.550**
***HEI***												**1**

* bolded values are different from zero with a significance level of alpha = 0.05.

**Table 4:** HQ scores obtained for the studied water samples if consumed.

	Al	As	Cr	Cd	Cu	Mn	Ni	Pb	Zn
**1**	0.45	0.06	2.2 × 10^−4^	5.4 × 10^−3^	2.7 × 10^−3^	5.0 × 10^−4^	1.0 × 10^−2^	6.2 × 10^−2^	1.7 × 10^−2^
**2**	0.07	0.08	2.0 × 10^−5^	5.4 × 10^−3^	1.3 × 10^−3^	0.0	4.5 × 10^−3^	2.6 × 10^−2^	8.7 × 10^−3^
**3**	0.41	0.04	3.4 × 10^−5^	9.7 × 10^−3^	3.5 × 10^−3^	0.0	2.7 × 10^−3^	5.6 × 10^−2^	2.0 × 10^−3^
**4**	0.40	3.1 × 10^−3^	2.0 × 10^−5^	8.4 × 10^−2^	3.9 × 10^−3^	5.9 × 10^−4^	7.3 × 10^−3^	5.4 × 10^−2^	1.8 × 10^−3^
**5**	2.4	2.8 × 10^−3^	7.4 × 10^−5^	5.2 × 10^−3^	2.3 × 10^−3^	5.6 × 10^−2^	0.01	0.02	2.2 × 10^−3^
**6**	0.48	0.05	9.6 × 10^−5^	0.01	5.9 × 10^−3^	1.8 × 10^−2^	4.9 × 10^−3^	0.2 × 10^−2^	8.9 × 10^−3^
**7**	8.0 × 10^−2^	1.4 × 10^−3^	1.7 × 10^−5^	7.6 × 10^−3^	1.6 × 10^−3^	6.2 × 10^−4^	4.7 × 10^−3^	2.1 × 10^−2^	2.7 × 10^−3^
**8**	0.10	0.0	3.3 × 10^−5^	1.0 × 10^−2^	6.3 × 10^−2^	3.7 × 10^−2^	7.2 × 10^−3^	2.2 × 10^−2^	3.9 × 10^−3^
**9**	8.6 × 10^−2^	2.1 × 10^−4^	3.5 × 10^−5^	4.3 × 10^−3^	4.2 × 10^−3^	1.1 × 10^−3^	1.1 × 10^−2^	2.1 × 10^−2^	2.2 × 10^−3^
**10**	2.0	1.9 × 10^−3^	4.5 × 10^−5^	4.7 × 10^−3^	5.5 × 10^−3^	0.0	4.6 × 10^−3^	1.8 × 10^−3^	8.3 × 10^−4^
**11**	0.41	9.6 × 10^−4^	9.5 × 10^−5^	8.8 × 10^−3^	7.7 × 10^−3^	1.2 × 10^−3^	5.2 × 10^−3^	3.4 × 10^−2^	7.2 × 10^−3^
**12**	0.11	0.01	2.6 × 10^−5^	8.8 × 10^−3^	2.2 × 10^−3^	0.0	8.5 × 10^−3^	5.0 × 10^−2^	5.3 × 10^−3^

## Data Availability

Not applicable.
